# Study on the Dynamic Biological Characteristics of Human Bone Marrow Mesenchymal Stem Cell Senescence

**DOI:** 10.1155/2019/9271595

**Published:** 2019-04-04

**Authors:** Xiongbin Chen, Lu Wang, Jiyin Hou, Jin Li, Linbo Chen, Jieyu Xia, Ziling Wang, Minghe Xiao, Yaping Wang

**Affiliations:** ^1^Department of Anatomy and Histology and Embryology, Basic Medical College, Chengdu University of Traditional Chinese Medicine, 1166 Liutaida Road, Wenjiang District, Chengdu, Sichuan 610075, China; ^2^Laboratory of Stem Cells and Tissue Engineering, Chongqing Medical University, 1 Yixueyuan Road, YuZhong District, Chongqing 400016, China; ^3^Department of Histology and Embryology, Chongqing Medical University, 1 Yixueyuan Road, Yuzhong District, Chongqing 400016, China

## Abstract

**Objective:**

To preliminary explore the senescent dynamic changes of the bone marrow mesenchymal stem cells (BMMSCs) by human ageing and its possible mechanism.

**Methods:**

The bone marrows were harvested from healthy volunteers, and according to volunteers' age, these were divided into group A (≤25 years), group B (26-45 years), group C (46-65 years), and group D (>65 years). Totally, the bone marrows were extracted from the posterior superior iliac spine from volunteers under aseptic conditions. Diluted with isovolumic PBS, followed by centrifugation at 1 × 10^5^/cm^2^, cells were cultured in a 5% CO_2_ incubator at 37°C. After three passages, surface marker identification of hBMMSCs was tested by flow cytometry (FCM), oil red O staining was used to observe the ability of osteogenic differentiation, alkaline phosphatase (ALP) staining and the levels of osteocalcin (OST) in the supernatants were used to observe the ability of adipogenic differentiation, senescence-associated *β*-galactosidase (SA-*β*-Gal) staining was used to detect the senescent BMSCs, the ability of BMSC proliferation was detected by cell counting kit-8 (CCK-8), the distribution of the cell cycle was analyzed by flow cytometry (FCM), and malondialdehyde (MDA) content, total glutathione peroxidase, total antioxidant capacity, and total superoxide dismutase (SOD) activity was analyzed using enzymatic assay.

**Results:**

The BMSCs highly expressed CD73 and CD90, but lowly expressed CD34 and CD19/CD14. With age, osteogenic differentiation was markedly increased and audiogenic differentiation was significantly decreased. The number of SA-*β*-gal-positive cells was significantly increased, the proliferation ability of hBMMSCs declined, the BMSCs were held in the G1 phase, the MDA level of BMSCs was significantly increased, and total glutathione peroxidase, total antioxidant capacity, and SOD activity significantly declined.

**Conclusions:**

With age, the aging BMSCs were intensified; the mechanism may be related to oxidative damage mediated aging-related pathways.

## 1. Introduction

Bone marrow is the most important hematopoietic organ and is composed of hematopoietic cells and bone marrow mesenchymal stem cells (BMMSCs) at different stages of development [1]. BMMSCs are nonhematopoietic cells and have multipotent differentiation potential. In addition, BMMSCs are the main component of the hematopoietic inductive microenvironment (HIM), where hematopoietic stem cells (HSCs) lodge, proliferate, differentiate, and are regulated, and they can also reflect the functional status of a hematopoietic inductive microenvironment and maintain the steady state [2]. In the hematopoietic inductive microenvironment, hematopoietic stem cells generate specific progenitor cells and the self-renewal divisions necessary to sustain themselves; those functions all associate with HIM. It has been observed for a long time that with advancing age, the number of HSCs decreases, which leads to deficiency in hematopoiesis and immunity in company of diseases related with aging. Our recent studies have shown that, with increasing age, the antioxidant capacity of HSCs remarkably decreases and superoxide accumulates, finally causing HSC senescence [3]. As the core component of HIM, as to whether BMMSCs can also manifest age-related senescence like HSCs can, there have been few reports concerning this. Thus, to better elucidate the underlying mechanism(s) in age-associated BMMSCs, here we investigated the effects of age in BMMSCs by humans.

## 2. Materials and Methods

### 2.1. Ethical Issues

The study protocol was reviewed and approved by the Ethics Committee of the First Affiliated Hospital of Chongqing Medical University. All participants were informed about the research purposes, procedures, and their rights. They were also assured that their information would be kept strictly confidential, and they signed informed consent forms.

The bone marrow samples were obtained from the ilia of a normal patient diagnosed without hematological diseases in clinic and who has not been admitted in the Department of Hematology of First Affiliated Hospital of Chongqing Medical University.

According to the patients' age, they were divided into 4 groups: group A including 10 patients aged 15 to 25 years old, group B for 12 patients aged 26 to 45 years old, group C for 15 patients aged 46 to 65 years old, and group D for 12 patients aged 66 to 85 years old.

### 2.2. Reagents

Foetal bovine serum (FBS), Dulbecco's modified Eagle's medium (DMEM), penicillin/streptomycin (P/S), fluorescein isothiocyanate (FITC) anti-CD34, FITC anti-CD14, FITC anti-CD19, phycoerythrin (PE) anti-73, PE anti-CDHLA-DR, PE anti-CD90, and PE anti-CD11b were obtained from BD Biosciences (Heidelberg, Germany). Osteogenic differentiation medium and adipogenic differentiation medium were obtained from Shenzhen Baien Biological Technology Co. Ltd. (Shenzhen, Guangdong, China). The SOD, TGP, and MDA kits were purchased from Nanjing Jiancheng Bioengineering Institute (Nanjing, Jiangsu, China). The IL-2 kit, TNF-*α* kit, IL-6 kit, BCA kit, and SA-*β*-gal staining kit were purchased from Beyotime Institute of Biotechnology (Shanghai, China). The CCK-8 kit was purchased from Shanghai Qihan Biological Technology Co. Ltd. (Shanghai, China).

### 2.3. hBMMSC Isolation and Culture

Human bone marrow mesenchymal stem cells (hBMMSCs) were derived from bone marrow harvested from the pelvis of healthy donors (15–85 years old) undergoing osteotomy for reasons other than metabolic disorders at the First Affiliated Hospital of Chongqing Medical University. The patients were informed about the sample collection and signed informed consent forms. The bone marrow was collected in MSC medium containing 0.01% of penicillin/streptomycin and 0.1% heparin. Bone marrow mononuclear cells (BMNCs) were isolated by density gradient centrifugation at 800 *g* for 20 minutes using lymphocyte separation medium. After being washed with PBS, the remaining cells including the marrow cells were incubated in flasks containing Dulbecco's MEM (DMEM), 1% penicillin + streptomycin, L-glutamine, and 10% foetal bovine serum (FBS) at 37°C in 5% CO_2_ for 72 h. Then, nonadherent cells were removed and the medium was changed weekly until cells were confluent. Then, the collected 3rd-generation hBMMSCs were used in the following experiments.

### 2.4. Characterization of hBMMSC Surface Antigens

Flow cytometry (FCM) was performed on hBMMSCs that were stained for CD73, CD34, CD14, CD19, CDHLA-DR, and CD90. The following antibodies specific for human molecules were used: PE-CD73, FITC-CD34, FITC-CD14, PE-CD90, FITC-CD19, PE-CDHLA-DR and PE-CD11b.

### 2.5. Osteogenic Differentiation

To induce osteoblastic differentiation, hBMMSCs were seeded at a density of 2.5 × 10^4^ cells/well in a 24-well plate with osteogenic induction medium at 37°C in 5% CO_2_ for 12 days, and the induction medium was changed for 4 days. The induction medium comprised of 10% FBS and 10% osteoblastic differentiation medium additive. For alkaline phosphatase (ALP) staining, cells were fixed with 4% paraformaldehyde and stained by calcium cobalt staining assay kit. ALP and osteocalcin (OCN) levels were measured by enzyme-linked immunosorbent assay (ELISA) according to the kit manufacturer's instructions.

### 2.6. Adipogenic Differentiation

To induce adipogenic differentiation, hBMMSCs were seeded at a density of 2.5 × 10^4^ cells/well in a 24-well plate with adipogenic induction medium for 12 days at 37°C in 5% CO_2_ and the induction medium was changed for 4 days. The induction medium comprised of 10% FBS and 10% adipogenic differentiation medium additive. Lipid droplets in the BMMSC cytoplasm were detected by oil red O staining.

### 2.7. Senescence-Associated *β*-Galactosidase (SA-*β*-gal) Cytochemical Staining

The hBMMSCs were collected at the 3rd generation and seeded at a density of 5 × 10^4^ cells/well in a 6-well plate with BMMSC medium and incubated for 4 days at 37°C in 5% CO_2_. SA-*β*-gal staining was performed according to the manufacturer's instructions.

### 2.8. CCK-8 Cell Proliferation Assay

The hBMMSCs were collected at the 3rd generation and inoculated onto a 96-cell/well plate at a density of 5 × 10^3^ and incubated for three days. At 0, 24, 48, 72, 96, 120, 144, and 168 h after hBMMSCs inoculating, the CCK-8 solution was added 3 h prior to the end of incubation. Cell viability was measured with a spectrophotometer at an absorbance of 450 nm.

### 2.9. Detection of Oxidation-Associated Biomarkers

At three passages, the hBMMSCs were collected and lysed in an ice bath for 30 min. After centrifugation (12000 rpm, 4°C, 30 min), the supernatant was collected. Total antioxidative capacity (TAC), SOD activity, total glutathione peroxidase (TGSH-PX), and MDA content (Beyotime Institute of Biotechnology, Shanghai, China) were detected by chemical colorimetric analysis according to the manufacturer's instructions.

### 2.10. Cell Cycle Analysis

The hBMMSCs were collected at a density of 1 × 10^6^ cells/ml per tubes and fixed with 70% ethanol for 12 h. After centrifugation, the supernatant was discarded, and incubation with 100 *μ*l pancreatic ribonuclease was performed at 37°C for 30 minutes. Then, propidium iodide staining was added and detected by flow cytometry.

### 2.11. Cell Apoptosis

The hBMMSCs were collected. Briefly, 1 × 10^6^ cells were incubated in PBS buffer mixed with propidium iodide and Annexin V, then hatched for 15 min in 37°C with the light ray blocked. Flow cytometry was used to detect the apoptosis rate.

### 2.12. Inflammatory Cytokine and Stem Cell Factor (SCF) Detection in hBMMSCs by ELISA

The supernatant of the cell culture medium at the 3rd generation was collected, and the levels of inflammatory cytokines (IL-2, IL-6, and TNF-*α*) as well as SCF were tested by ELISA according to the kit manufacturer's instructions.

### 2.13. RNA Extraction and Real-Time Quantitative RT-PCR

The hBMMSCs of each group were collected after culturing the 3rd generation. Total RNA was isolated using TRIzol reagent (Invitrogen, USA) according to the manufacturer's protocol. First-strand cDNA was created by TaqMan RT reagents (Applied Biosystems, USA). Quantitative real-time PCR was performed using SYBR Green Supermix (Bio-Rad) on an iCycler Real Time Detection System (cfx96, Bio-Rad). Expression levels of mRNA were normalized against GADPH and analyzed by the comparative cycle threshold method. The means ± SD of three independent experiments were determined. The PCR primers used are provided in the supporting information.

### 2.14. Statistical Analysis

Data were analyzed by one-way ANOVA using SPSS Version 18.0 software. Differences were considered significant at *P* < 0.05. Different letters “a–d” represent a significant difference between intergroups, and same letters “a–d” represent no difference between intergroups (*P* > 0.05).

## 3. Results

### 3.1. Characterization of Cultured hBMMSCs

**Figure 1:**
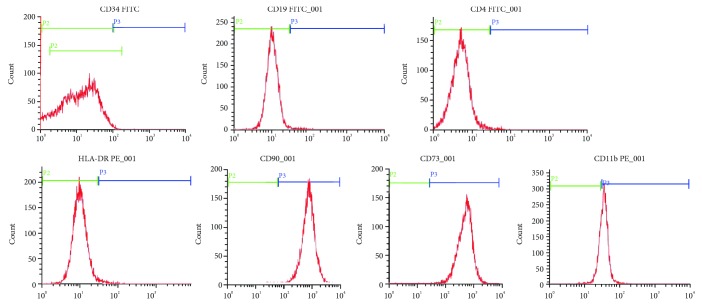
The expression profiles of BMMSC surface markers in humans determined by FACS.

For immunophenotyping of cultured hBMMSCs, flow cytometry showed that markers are positive CD73, CD90 and negative for CD34, CD19, CD14, and HLA-DR. The results demonstrated that the cultured cells were typical hBMMSCs (Figure 1).

### 3.2. Osteogenic Differentiation

**Figure 2:**
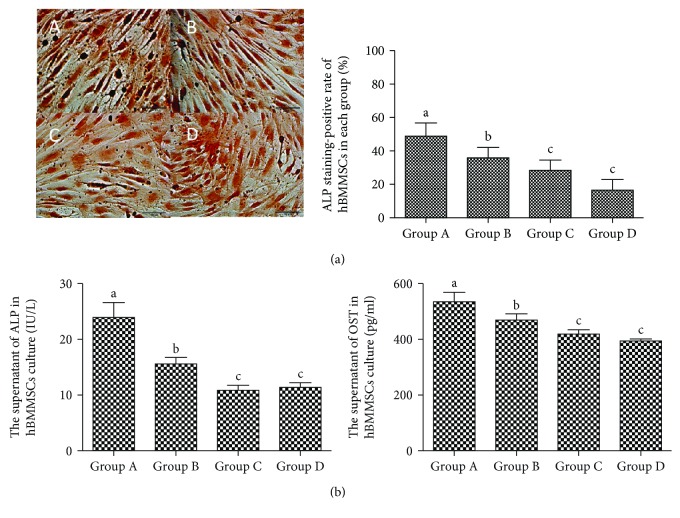
Positive ALP staining in different groups of hBMMSCs (×400). (a) Representative micrographs depict morphology of ALP staining-positive cells and the percentage of ALP staining-positive cells in hBMMSCs (%). Scale bars indicate 100 *μ*m. Positive cells are dyed in black. A for group A, B for group B, C for group C, and D for group D. (b) The ALP and OST content in culture supernatant of different hBMMSCs. Different letters of a–c on the panels of group A, group B, group C, and group D mean that, compared with each other, there is a significant difference between intergroups (*P* < 0.05). Same letters on the panels of group A, group B, group C, and group D mean that compared with each other, there is no significant difference between intergroups (*P* > 0.05).

In ALP staining, the positive cells were stained in black granules in the cytoplasm. The supernatant of the cell culture was collected, and the levels of ALP as well as OST in each group were measured by ELISA (Beyotime Institute of Biotechnology, Shanghai, China) according to the kit manufacturer's instructions. The result showed that the osteogenic differentiation potential and the content of ALP and OST decline with age (Figure 2).

### 3.3. Adipogenic Differentiation

**Figure 3:**
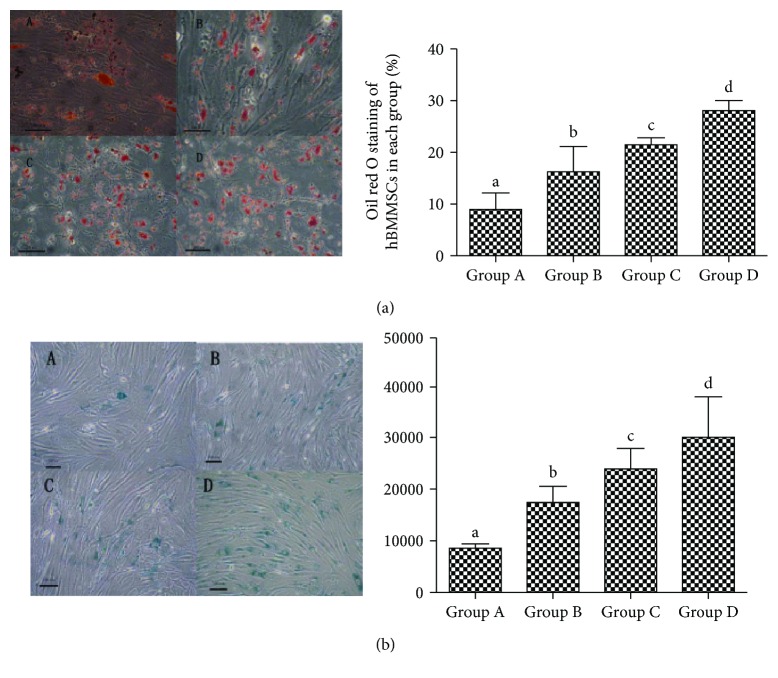
The oil red O staining and senescence-associated beta-galactosidase staining in different groups of hBMMSCs (×200). (a) The oil red O staining and percentage of oil red O-positive cells in hBMMSCs; positive cells are dyed in red. (b) Senescence-associated beta-galactosidase staining and the IOD of senescence-associated beta-galactosidase staining of hBMMSCs in different groups; positive cells are dyed in blue. Bar = 100 *μ*m. A for group A, B for group B, C for group C, and D for group D. Different letters of a–d on the panels of group A, group B, group C, and group D mean that compared with each other, there is a significant difference between intergroups (*P* < 0.05). Same letters on the panels of group A, group B, group C, and group D mean that compared with each other, there is no significant difference between intergroups (*P* > 0.05).

In oil O staining, the positive cells were stained in red in the cytoplasm. Oil O staining revealed a significant increase in each group of hBMMSCs with age (Figure 3(a)).

### 3.4. The Change in SA-*β*-Gal Staining in the hBMMSCs with Age

In SA-*β*-gal staining, the positive cells were stained in blue with blue granules in the cytoplasm and the negative cells were not stained. The results revealed a significant increase in integrated optical density (IOD) of hBMMSCs with age (Figure 3(b)).

### 3.5. hBMMSC Proliferation by Aging

**Figure 4:**
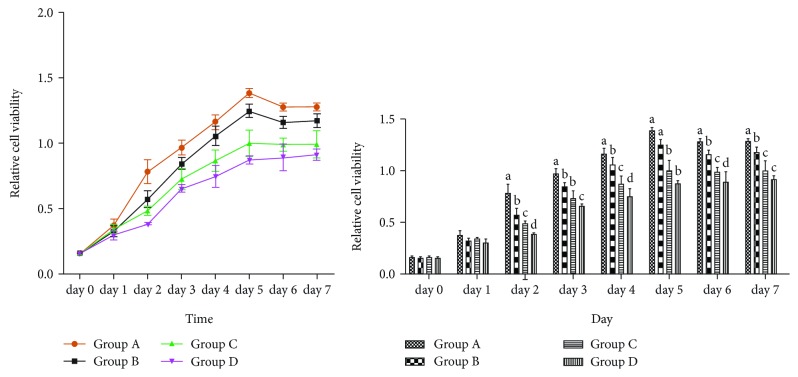
The growth curve and absorbance of hBMMSCs in different groups. Different letters of a–d on the panels of group A, group B, group C, and group D mean that compared with each other, there is a significant difference between intergroups (*P* < 0.05). Same letters on the panels of group A, group B, group C, and group D mean that compared with each other, there is no significant difference between intergroups (*P* > 0.05).

Since two days, the proliferation of hBMMSCs revealed an inhibition from group A to group D, illustrating that the ability of proliferation of hBMMSCs gradually decreased by aging (Figure 4).

### 3.6. The Cell Cycle of hBMMSCs by Aging

**Figure 5:**
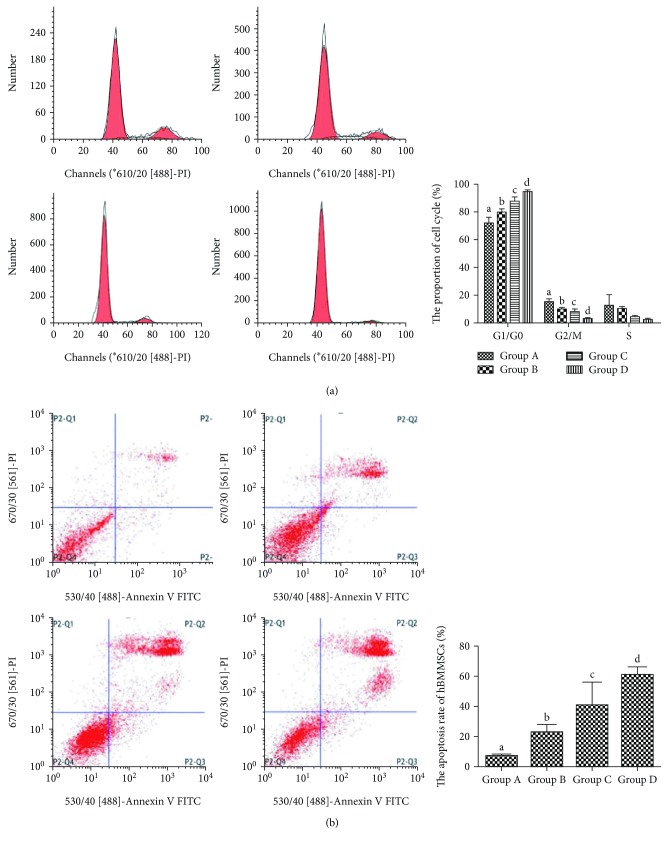
The cell cycle distribution and apoptosis rate of hBMMSCs in different groups. (a) Flow cytometry graphs and the cell cycle distribution of hBMMSCs in different groups. (b) Flow cytometry graphs and the apoptosis rate of hBMMSCs in different groups. Q1 represents necrosis, Q2 represents late apoptosis, Q3 represents early apoptosis, Q4 represents normal cells. A for group A, B for group B, C for group C, and D for group D. Different letters of a–d on the panels of group A, group B, group C, and group D mean that compared with each other, there is a significant difference between intergroups (*P* < 0.05). Same letters on the panels of group A, group B, group C, and group D mean that compared with each other, there is no significant difference between intergroups (*P >* 0.05).

Flow cytometry showed that the cell cycle phase distribution of hBMMSCs was arrested in G0/G1, and the proportion of the S phase reduced with age (Figure 5(a)).

### 3.7. Cell Apoptosis of hBMMSCs by Aging

Flow cytometry showed that the cell apoptosis rate of hBMMSCs was elevated significantly with age (Figure 5(b)).

### 3.8. Superoxide Damage and the Anti-superoxide Ability in hBMMSCs

**Figure 6:**
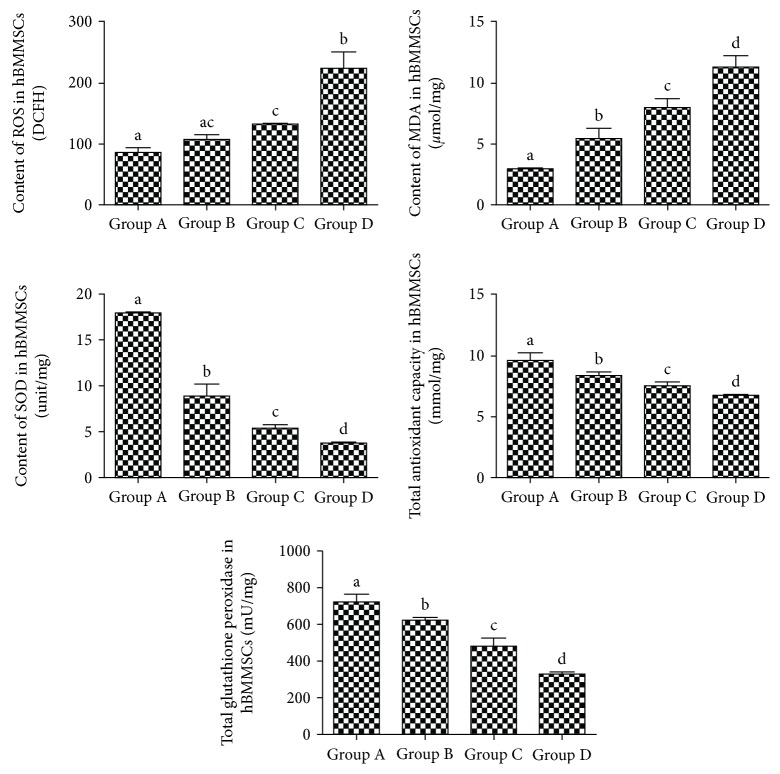
Superoxide damage and the anti-superoxide ability of hBMMSCs in different groups. Total contents of ROS, MDA, and SOD; total antioxidant capacity; and total glutathione peroxidase in different groups of hBMMSCs. A for group A, B for group B, C for group C, and D for group D. Different letters of a–d on the panels of group A, group B, group C, and group D mean that compared with each other, there is a significant difference between intergroups (*P* < 0.05). Same letters on the panels of group A, group B, group C, and group D mean that compared with each other, there is no significant difference between intergroups (*P* > 0.05). Letters “ac” on the panel of group B mean that compared with group A and group C, there is no significant difference between intergroups (*P* > 0.05).

From groups A to D, the activities of SOD, TGSH-PX, and total antioxidative capacity decreased significantly and the MDA content increased significantly in hBMMSCs (Figure 6).

### 3.9. Inflammatory Cytokine and SCF Levels of hBMMSCs by Aging

**Figure 7:**
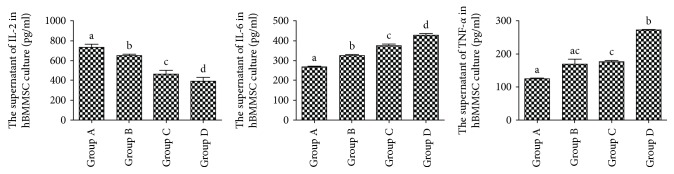
Inflammatory cytokine secretion by hBMMSCs in different groups. Different letters a–d on the panels of group A, group B, group C, and group D mean that compared with each other, there is a significant difference between intergroups (*P* < 0.05). Same letters on the panels of group A, group B, group C, and group D mean that compared with each other, there is no significant difference between intergroups (*P* > 0.05). Letters “ac” on the panel of group B mean that compared with group A and group C, there is no significant difference between intergroups (*P* > 0.05).

From groups A to D, the levels of IL-6 and TNF-*α* significantly increased, while IL-2 and SCF significantly decreased (Figure 7).

### 3.10. mRNA Expression of p53 and p21^Cip1/Waf1^

**Figure 8:**
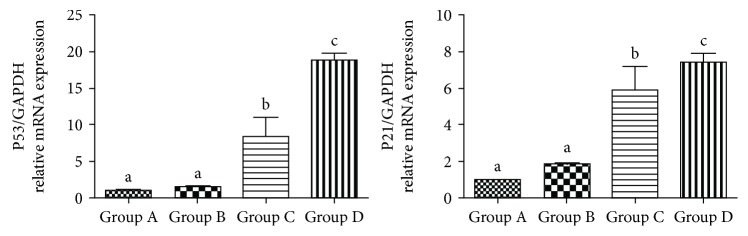
The expression of mRNA in different groups of hBMMSCs. Different letters a–d on the panels of group A, group B, group C, and group D mean that compared with each other, there is a significant difference between intergroups (*P* < 0.05). Same letters on the panels of group A, group B, group C, and group D mean that compared with each other, there is no significant difference between intergroups (*P* > 0.05).

The p53-p21^Cip1/Waf1^ pathway is considered as an essential way to regulate cell senescence. By qRT-PCR analysis, it showed that mRNA expressions of p53 and p21^Cip1/Waf1^ were significantly increased in the hBMMSCs from groups A to D (Figure 8).

## 4. Discussion

Bone marrow mesenchymal cells have been considered as a good support function for sustaining hematopoietic stem cells (HSCs), and they have been regarded as a promising seed due to their capacity to differentiate into osteoblasts and so forth [4]. Our previous study had demonstrated that HSCs undergo progressive morphologic and functional changes in natural aging. However, changes in BMMSCs with aging are still unknown. Thus, to better illuminate the underlying changes of aging in BMMSCs, we investigated the effects of aging on human BMMSCs in vitro.

The hBMMSCs isolated from human bone marrow and passed third. Currently, the Mesenchymal and Tissue Stem Cell Committee of the International Society for Cellular Therapy had proposed criteria to define BMMSCs [5]. One of the standards is that BMMSCs' surface must express CD73 and CD90 and lack the expression of CD14 or CD11b or CD19 and HLA-DR. In this study, we used various sets of surface markers to identify the purity of BMSCs. The results demonstrated that hBMMSCs highly expressed CD73 and CD90 and moderately expressed CD11b, while it was negative for CD19, CD14, and HLA-DR, consistent with standard criteria. In addition, another consensus opinion on BMMSCs was that it must differentiate to osteoblasts and adipocytes in vitro. However, evidence had suggested that the hBMMSCs' differentiation capacity of osteogenesis declines with age, adipogenic differentiation potential increases with aging [6]. In contrast, in our study, we also found that with advancing aging, the capacity of osteoblasts and the level of osteocalcin (OCN) and alkaline phosphatase (ALP) decrease while the capacity of adipocytes increases in hBMMSCs. The observation above indicates that there may be an unknown mechanism in lineage determination of hBMMSCs with aging.

During the HSCs' natural aging, the ability of differentiation and self-renewal decline result in reduction in the number of HSCs and a rise in myeloid leukemia and aplastic anemia, which increases with aging [7]. BMMSCs could provide a microenvironment for the HSCs that settled [8], and it is shown that HSCs function similarly with BMMSCs which consisted a niche with another cells [9, 10]. This tendency suggests that BMMSCs produced senescence-like alterations with aging. Senescence-associated markers SA-*β*-galactosidase could stain positive cells in blue [11]. In this study, we observed that the number of positive senescence cells increased with aging as well as the IOD. In addition, we also found that proliferation capability decreased with aging. While the cell cycle and apoptosis analysis by flow cytometry and results showed that hBMMSCs displayed G1 phase arrests and an apoptosis rate increase with aging, the cell cycle proportion of the G1/G0 phase increased and that of the S phase decreased. From all the above results, we speculated that in the process of aging, there were senescent changes in hBMMSCs with aging. It indicated that age was a major cause of aging for hBMMSCs.

Aging had been confirmed to be associated with chronic inflammation and to play an important role in several age-related diseases such as cancer, atherosclerosis, and osteoarthritis [12]. Recent studies have shown that when the cells occurred to be aging, there was a striking increase in inflammatory cytokines [13–15]. In addition, IL-2 connected with the immune system and low level of IL-2 could cause immunosenescence [16]. In this study, we also observed that inflammatory cytokines, such as IL-6 and TNF-*α*, increased and the level of IL-2 decreased with age.

Now, there are many kinds of theories about cell senescent mechanism, including oxidative stress and free radical theory. The free radical theory showed that free radicals could induce cell DNA damage and such damage is of central importance to triggering cell senescence [17]. Reactive oxygen species (ROS) is one of the key factors of cellular senescence, and several evidences indicated that a high level of ROS could trigger senescence in in vitro culture and damage DNA, proteins, and lipids in organisms [18, 19]. MDA, the product of lipid peroxidation when free radicals attack polyunsaturated fatty acids in the biomembrane, has been used as a biomarker for oxidative stress [20]. The content of MDA can directly reflect the level of lipid oxidation and indirectly reflect oxidative stress in cells [21]. In this study, we found that the content of MDA and ROS in cells increased with aging; this demonstrated that superoxide damage of hBMMSCs increased significantly. Conversely, the antioxidant ability of organisms can neutralize ROS and prevent against MDA injury, such as superoxide dismutase (SOD), total antioxidant capacity, and total glutathione peroxidase. In the present study, we also found that SOD, total antioxidant capacity, and total glutathione peroxidase in the hBMMSCs were decreased with increasing age. From the above study, we speculate that in the process of natural aging, because of accumulation of free radicals, it can attack the mitochondria in the biomembrane and cause mitochondria dysfunction, which may eventually exceed the capacity of cellular antioxidant defense and cause cell senescence. Hence, we believe that oxidative stress plays an important role in cell aging.

Our previous research showed that the number of hematopoietic stem cells (HSC) in the bone marrow decreases, their oxidative stress increases, and antiaging ability was lost gradually when the body got older; in this research, we also found that hBMMSCs showed the same aging phenomenon consistent with HSC aging. A variety of stresses can trigger senescent pathways such as the p53-p21^Cip1/Waf^ pathway. These stresses include intracellular and extracellular stresses. Oxidative stress is an important senescent stress; our previous study showed that the p53-p21^Cip1/Waf^ pathway was activated in HSC senescence. According to this study, the expression of senescence-related mRNA (p53 and p21) in the hBMMSCs was significantly elevated with age. Thus, it implied that the bone marrow gradually undergoes senescence as we grew older. And it may also indicate that the age-related changes in the bone marrow related with hematological diseases, such as anemia, increased propensity for myeloproliferative neoplasms (MPNs), decreased the immune function, increased leukemia, and other cancer incidences. It also enlightened us that maybe we can use drug intervention to improve this antiaging capability to reduce the incidence of these diseases. These are also what we are most interested in and that we will to continue to research in the future.

## Data Availability

The data used to support the findings of this study are available from the corresponding author upon request.
